# Notes on Bedford Springs, and the Diseases in Which a Resort to Them Is Useful

**Published:** 1852-06

**Authors:** Caspar Morris

**Affiliations:** Philadelphia


*Notes on Bedford Springs, and the Diseases in which a resort to them is useful.* By CASPAR MORRIS, M., D. of Philadelphia.

Physicians in all our great cities, and in many country places, are annually consulted by those under treatment for chronic diseases as to the *best* place to which they shall resort for the benefit of change. Watering and bathing places are too often recommended without any discrimination, under the vague impression that mere change of scene and change of air, and relaxation from customary pursuits, are all-efficient agents in the restoration of healthful action to the organs, and sensations to the body and mind. The power of these influences is undoubtedly very great, and should never be overlooked in our estimate of the value of causes; yet they should not be permitted to usurp the position of chief agents, much less to exclude from our regard others equally operative. The *deleterious* influence of change of water is universally recognized; every ailment experienced during travel, andmany subsequent inconveniences, are, even in popular language, ascribed to it. Nor is this at all irrational. Next to the air we inspire, the water we drink is the most essential to our existence of all the influences by which it is supported. Taken into the stomach in quantities varying in different persons, but in all vastly greater than the solid aliment, and passing directly by endosmosis into the circulating fluids, quantity and quality alike must produce a powerful effect on the blood, and through this fluid on the actions of the several secerning organs which eliminate from the system, either the poisonous, extraneous or effete matters which are found in it. The value of springs impregnated with saline matters in larger proportions, or of a character different from those found in common water, has always been recognized; and many of the places frequented by the invalids of the old world at the present day, have been thus resorted to from the earliest periods of which records have been transmitted.

This testimony of experience is of great value, since nothing but truth abides the test of long and close scrutiny.

It is not, however, by experience only that the worth of remedies is to be determined, since we are all familiar with the observation, that ill observed facts make a fictitious experience. It is therefore always important to investigate the character of agents at the same time that we watch influences, that the knowledge derived from either source may reflect itself on that deduced from the other. Hence arises the necessity for chemical analysis, by which we are enabled to confirm or refute the claims set forth in behalf of any given water.

Among the many springs charged with mineral ingredients, which are scattered over our country, few have attracted more attention, or maintained their reputation longer, than those near the town of Bedford, in the county of the same name, in this State. Yet to the members of the profession in the city of Philadelphia, their merits are almost unknown, and but few, comparatively, of our citizens resort to them for that renovation of health they are so well calculated to promote. Unnoticed in the Dispensatory of Wood and Bache, and but slightly in the original work of Dr. Bell on Mineral Waters, our knowledge of them is limited to a brief tract read before the medical faculty of Pittsburgh by Dr. Church of that city, so far back as the year 1825. Having twice derived great benefit from a personal resort to the Springs, and frequently from the use of the water at home, I have thought it not improper at this season to invite the attention of the readers of the *Examiner* to the subject, in the hope that others may be induced to try their virtues.

Bedford County lies near the Southern boundary of the State of Pennsylvania, in the midst of the western spurs of the Alleghany mountains, and abounds in all the variety of soil and surface which belongs to mountainous districts; well cultivated and fertile valleys alternating with mountainous ridges. The town of Bedford lies on the southern fork of the Juniata; and about two miles from it, in a narrow valley, are the Springs. There are three routes by which it is approached from the eastward. The Baltimore and Ohio Railroad passing through the fertile farms of Washington County, Maryland, and the rugged scenery which is so celebrated at the place where the Shenandoah and Potomac divide the Blue Ridge, and then across the the valley of Virginia, carries passengers to Cumberland, from whence a rough stage road of thirty miles leads to the Springs. The Susquehanna Railroad from Baltimore, or the Columbia Railroad from Philadelphia, convey them to Harrisburgh, where they can either take the Central Railroad to Hollidaysburgh, following the course of the Juniata to that point, from which stages are also required to the Springs, the distance being about as great as that from Cumberland, and the stage road of the same character; or the Cumberland Valley railroad, passing through Carlisle to Chambersburgh, where it terminates, affords a very pleasant route. From Chambersburgh the distance is sixty miles, by good turnpike and comfortable stages. This road crosses all the mountain ridges, and presents a ride of unrivalled attraction to those who have strength to endure the fatigue, and taste to enjoy the delights of mountain scenery; good stopping places on the way present accommodations for those who are unable to accomplish the whole distance without rest. The town of Bedford itself, has no attractions, but good lodgings are to be had there by those who do not desire to stay at the springs, carriages passing to the Springs at all hours to take invalids there, or convey the water to them.

The natural beauty of the valley in which the springs are found is great, and the arrangements for the reception of visitors sufficiently convenient, though susceptible of great enlargement and improvement. The buildings are erected at the foot of one spur of a mountain which rises abruptly behind them to the west, while the valley is bounded on the eastern side by a similar hill which, clothed with wood of uncommon beauty, rises from the borders of the stream which waters the valley. It is at the foot of this hill the mineral spring is found, bursting from a fissure in lime-stone rock, and discharging, with but little fluctuation, a barrel of water per minute. Terraced walks are cut on this hill, leading to the summit, where a pavilion presents an opportunity for rest; while the view which it opens over the valley in which the town of Bedford lies, affords an ample recompense for the toil of the ascent. To those fond of investigating the mysteries of nature, the fossil remains, found abundantly in the limestone and red sandstone formations, present constant attractions of unusual interest; while fishing and shooting furnish amusement of a healthy kind to those of less scientific tastes. These accessory circumstances are important, yet the object of this notice is rather to draw attention to the qualities and influence of the waters. Within a very small area are to be found one spring of pure slate water; another impregnated with hydro-sulphuric acid; a very copious spring of mountain limestone water, and that which gives especial value to the spot, "the mineral spring." This diversity in the water is easily explained, from the geological character of the valley, lying, as Mr. Rogers, the State Geologist, expresses it, on the "dislocated side of a synclinal trough or basin in the strata, which are there steeply uptilted, and hence there is probably a very extensive surface drainage." The red sandstone, the slate and the fossiliferous limestone, are here all brought to the surface, and from the different nature of these strata the various characters of the water here thrown out are derived.

I have been permitted to see the MSS. notes of the analysis of the Sulphur Spring made by the Rev. Dr. Humphreys, of St. John's College, Annapolis, which indicate a very decided character of the same kind with that of the Sulphur Springs of other localities. I have, however, never tested its virtues, and am not competent to determine its merits.

The water of "the Mineral Spring," as it is called, was analysed by Dr. Church, of Pittsburg, in the year 1825; who gave the result to the public through the medium of a Communication to the Pittsburg Medical Society. Dr. C. says, "The temperature of the water was 58° F., while that of the atmosphere was 70°," and gives the specific gravity as 1029, and then details the various means to which he resorted to test the saline ingredients. According to the analysis of Dr. C. "a quart of water evaporated to dryness, gave thirty-one grains of residuum. The same quantity of water treated agreeably to the rule laid down by Westrumb, contained eighteen and a half cubic inches of carbonic acid gas. The residuum treated according to the rules given by Dr. Henry in his system of Chemistry, gave the following result:

| | |
|---|---|
| Sulphate of Magnesia, - - - - - | 20 grs. |
| Sulphate of Lime, - - - - - | $3\frac{3}{4}$ grs. |
| Muriate of Soda, - - - - - | $2\frac{1}{2}$ grs. |
| Muriate of Lime, - - - - - | $\frac{3}{4}$ grs. |
| Carbonate of Iron, - - - - - | $1\frac{1}{4}$ grs. |
| Carbonate of Lime, - - - - | 2 grs. |
| Loss, - - - - - - - - - | $\frac{3}{4}$ grs. |

Making a total of 31 grains; to which must be added $18\frac{1}{2}$ inches of carbonic acid gas."

The analysis thus described was made twenty-seven years since at the Springs. A quantity of the water was sent to me last autumn, in well cleansed bottles, closely corked and sealed, and the following is a memorandum of the result of an analysis made by my son, J. Cheston Morris, in the laboratory of Professor Booth, and under his superintendence:

"One pint of the water evaporated at 240° F., yields of solid residuum - - - - - grs. 22.201

| | | | |
|---|---|---|---|
| $CaO, CO_2$ | Carbonate of Lime - | 2.120 | |
| $CaO, SO_3$ | Sulphate of Lime - | 11.274 | |
| $MgO, SO_3$ | Sulphate of Magnesia - | 3.974 | |
| $(Al_2O_3+Fe_2O_3)\ SO_3$ | Sulphates of Alumina & Iron | 1.280 | |
| $NaO, SO_3$ | Sulphate of Soda - - | 3.092 | |
| $NaCl$ | Chloride of Sodium - | 0.343 | |
| $SO_3$ | Free Sulphuric Acid - | 0.128 | |
| $SiO_3$ &c. | Organic matter and Silica | *trace* | 22.201 |

"In the estimation of the iron as a persulphate, there is probably an error, which would, if corrected, increase slightly the amount of free sulphuric acid. The quantity of iron and alumina is so small as to render it almost impossible to separate them correctly."

It will of course, be observed that the results of these analyses differ materially. The absence of carbonic acid gas in the latter, is readily accounted for by the presumption that it escaped; but while Dr. C. found but thirty-one grains of solid residuum in the quart, the analysis made by my son gives not less than forty-four grains. The relative proportion of the several ingredients is also materially different, the sulphate of magnesia giving place to the sulphates of lime and soda as the predominating ingredients, though the actual quantity of the sulphates of magnesia and soda taken together in the quart of water is nearly two-thirds that of the former, supposed by Dr. Church to exist in the same quantity of the water.

Every one must at once admit that a water so highly charged with saline ingredients must produce a decided impression on the stomach and bowels. Even admitting that there is no imbibition of the saline matter into the blood, so large a quantity passing over the mucous membrane of the alimentary canal, must stimulate the peristaltic action by mere mechanical irritation. But if, as I believe, it passes directly into the circulating fluid, without any change, and the saline materials, mingling with those which already exist in the serum of the blood, are presented to the emunctories of the liver, the kidneys and the skin, to stimulate them to an excess of action, we must admit that the result must be a strong impression, either for good or ill, as the condition of the patient may be adapted to its use or not. It is, I know, a common remark that if the benefits derived from a residence at these Springs, be due to the peculiar ingredients held in solution by them, we ought to be able to produce the same results by the employment of the same salts dissolved in the same quantity of fluid artificially. To this it is only necessary to reply, that we are never able by synthesis to produce a compound in all respects analogous to that from which a given result is produced by chemical analysis. The art of the chemist may ascertain the amount of solid matter, but cannot decide positively as to the precise relations it occupies before being subjected to his experiments; much less can he determine that it shall combine in precisely the same manner. The sources from which the saline materials are derived, are quite unknown, as is also the manner of their introduction into the water. One of the many interesting subjects for investigation, presented by geology, is the question whence these saline impregnations are supplied. If, as is generally presumed, by drainage of water through deposits of the material, how is the quantity dissolved kept so uniform from age to age, and how are the relative proportions of the different ingredients preserved without variation ?

Leaving these points, however, for future investigation, permit me to solicit the attention of your medical readers to the nature of the cases in which they may anticipate benefit from these waters. First in importance and frequency I should place those suffering from that deranged condition of the digestive organs which is so common among persons of sedentary habits and constant devotion to business. What the precise pathological condition of such cases is, it may be difficult to determine. It is generally believed that the functions of the liver are imperfectly performed. The stomach is enfeebled, and the nervous system exhausted. The food is imperfectly digested, the bowels are disposed to constipation, and the mind loses its natural vigor. The anxieties of the counting-house, the mental application of the divine, the physician and the lawyer, create numberless cases of this kind annually ; and though the relaxation and amusements of the bathing places, and the invigorating influence of sea atmosphere may afford some relief, these do not act so directly on the organs whose functions are impaired, and cannot, therefore, produce so useful an impression as is received at the Springs.

The sensible action of the water of "the Mineral Spring" at Bedford, is on the kidneys, producing very prompt and profuse diuresis ; on the skin, giving rise to very free perspiration ; and on the bowels, causing gentle catharsis. It will thus be evident that all the emunctories are stimulated to increased activity ; the discharges are copious, and yet not only is no debility induced, but there is an actual increase of vital force, in proportion to this activity. I have myself twice gone to Bedford, so prostrated as scarcely to endure the fatigue of the journey, and wholly disqualified for all exertion, and have in both instances returned, at the end of a fortnight or three weeks, restored to my wonted power of labor; and have witnessed similar results in the cases of friends and patients. This increase of energy cannot be justly attributed to the mere catharsis and diuresis, disgorging the portal circulation, and thus promoting digestion and assimilation; though, undoubtedly, much is due to this cause.

Though the quantity of iron is so small that it can scarcely be supposed capable of producing any very decided impression, it is possible that the sulphuric acid may add intensity to its action. Many persons, on partaking of the water, are sensible of a feeling in the head very similar to that produced by the mineral tonics, and which renders it impossible to continue to use it, unless the system is relieved by purging. In such cases, the use of a blue pill at night, and the addition of a small quantity of sulphate of magnesia to the water taken in the morning, removes all the unpleasant impression. The degree of benefit derived from the use of the water, is much influenced by the manner of its employment. The patient should rise early, and, before dressing, drink half a pint of the water in his room. After dressing he should resort to the spring, drink a tumbler of the water, and then walk smartly, either in the pavilion or on the walks provided for the purpose in the vicinity of the spring, for at least ten minutes, at the end of which time another tumbler is to be taken, followed by a similar walk. This process should be repeated, alternating the draughts and walks, until five tumblers of the water are drunk, in the course of an hour or an hour and a half. Those who are able should go to the top of the mountain, to which a well graded walk of a mile, leads; seats are provided at proper distances, on which those may rest who are not able to accomplish the whole distance. Two hours should be occupied in the drinking and walking before breakfast, during which time the skin and kidneys will pour forth an amount of fluid proportioned to the quantity which has been swallowed, and these secretions should be promoted by exercise adapted to the strength of the invalid. The quantity mentioned will generally occasion some three or four watery evacuations from the bowels, of a bright yellow color, without pain or exhaustion. Should this not occur during the two hours following breakfast, another glass should be swallowed before dinner; and in case the bowels should still resist the influence of the water, a dose of blue pill should be taken at bed-time, followed, in the morning, either by calcined magnesia, or the addition of epsom salts to the water. I have never known the water to prove violently or painfully active, but in one person. In such an event the use of it should be suspended. I have always kept a supply of the water at home; and have always found the use of it there remove that restless, irritable condition of the nervous system, caused by a slight disturbance of the digestive functions, which, in persons of what are called a bilious temperament, so often destroys the comfort of life. Like the draught of the fabled spring, it restores the spirits, and the capacity of enjoyment.

There is another large class of patients, whose diseases depend on the same causes, who derive equal benefit from the resort to the Bedford Springs—those who suffer from what is known as sick headache, in all its grades. An annual visit of a fortnight or three weeks, is found by many to diminish the frequency and intensity of these attacks, even where it does not entirely remove the cause. The same remark is applicable to those who suffer from the headache of irregular gout. In truth, so decided is the action of this water on the liver, that all the various forms of bilious derangement derive benefit from its use, unless there be some local inflammation. Cases thus complicated are always injured by it.

But while the class of patients thus far alluded to are found chiefly in our great cities, there is another of kindred character which is found in the miasmatic districts of the country; persons in whom the liver is either engorged or otherwise disabled for the proper performance of its functions, and who, with sallow complexion and languid appearance, find themselves unable to perform their accustomed duties. Such persons should always pass the last weeks of August and the month of September, in some situation free from the miasm which gives rise to remittent and intermittent fevers. Strange as it may appear, these diseases are unknown in the vicinity of Bedford. The temperature of the night is always so cool, that blankets are required on the beds, and often fire in the sitting rooms during the evening, even while the thermometer at mid day rises to 75° or 80°. The condensation of the moisture thus evaporated during the day by the coolness of the night, causes the valley to be filled by a dense fog every morning, which is deposited on the leaves of the trees which clothe the mountains, giving them their peculiar luxuriance of growth and beauty of foliage. From this well known character of the place, some have hastily inferred its liability to autumnal fever. Dr. Watson, who has resided there twenty years, and whose father had the entire medical charge of the town and neighborhood before him, assured me no case of intermittent or remittent fever had ever occurred under his observation. During one of my visits at the Springs, a young gentleman from Maryland, who was subject to constant recurrence of ague every few weeks, had one of his usual attacks, and I found on making enquiry at the stores where medicine was sold, that quinine was but little used, and received from all sources ample confirmation of the statement of Dr. Watson.

In the large class of cases known as anæmia, in which, from some unknown cause, the blood becomes vitiated, and the system consequently enfeebled, much good will be derived from the resort to Bedford. The quantity of iron in the water, though small, is in a condition to be absorbed and applied directly to the purpose for which it is needed, and its usefulness is enhanced by the cotemporaneous impression of the saline matter in the water, and the purging and diuresis.

The influence of this water on the health, has always been an ample compensation for the difficulty and fatigue of getting to the Springs. Railroads now facilitate the access, bringing it in fact within a few hours easy travel;—and whether we regard the healthfulness of the locality—the attractiveness of the scenery at the spot, in the vicinity and on the route, or the influence of the water itself, Bedford Springs present attractions to the valetudinarian, which cannot be surpassed by any part of the United States.

